# Changes in Meningococcal Strains in the Era of a Serogroup C Vaccination Campaign: Trends and Evolution in Belgium during the Period 1997–2012

**DOI:** 10.1371/journal.pone.0139615

**Published:** 2015-10-01

**Authors:** Wesley Mattheus, Germaine Hanquet, Jean-Marc Collard, Raymond Vanhoof, Sophie Bertrand

**Affiliations:** 1 Sections of Bacterial Diseases, Scientific Institute of Public Health, Brussels, Belgium; 2 Medical Epidemiologist, Health Care Knowledge Centre, Brussels, Belgium; The Australian National University, AUSTRALIA

## Abstract

**Background:**

Invasive meningococcal disease (IMD) is a major cause of bacterial meningitides and septicaemia. This study shows the results of the laboratory-based surveillance of IMD in Belgium over the period 1997–2012.

**Methods:**

The results are based on microbiological and molecular laboratory surveillance of 2997 clinical isolates of *N*. *meningitides* received by the Belgian Meningococcal Reference Centre (BMRC) over the period 1997–2012.

**Results:**

Serogroup B has always been a major cause of meningococcal disease in Belgium, with P3.4 as most frequent serotype till 2008, while an increase in non-serotypable strains has been observed in the last few years. Clonal complexes cc-41/44 and cc-269 are most frequently observed in serogroup B strains. In the late nineties, the incidence of serogroup C disease increased considerably and peaked in 2001, mainly associated with phenotypes C:2a:P1.5,2, C:2a:P1.5 and C:2a:P1.2 (ST-11/ET-37 clonal complex). The introduction of the meningococcal C conjugate vaccine has been followed by an 88% significant decrease in serogroup C disease from 2001 to 2004 nationally, yet sharper in Flanders (92%) compared to Wallonia (77%). Since 2008 a difference in incidence of serogroup C was observed in Flanders (0–0.1/100,000) versus Wallonia (0.1–0.3/100,000).

**Conclusion:**

This study showed the change in epidemiology and strain population over a 16 years period spanning an exhaustive vaccination campaign and highlights the influence of regional vaccination policies with different cohorts sizes on short and long-term IMD incidences.

## Introduction

*Neisseria meningitidis* is a major cause of bacterial meningitis and septicaemia worldwide. This bacteria is an obligate commensal of humans which normally colonizes the respiratory tract, without causing invasive disease, a phenomenon known as carriage [1;2]. Despite of high rates of meningococcal carriage, invasive meningococcal diseases (IMD) has become rare in industrialized countries the last decade, with annual incidence rates that vary from 1 to 3 cases per 100,000 individuals. Nevertheless, despite appropriate treatment, the case-fatality rate varies from 5% to 15% due to fulminant septicaemia frequently resulting in sequelae [2;3].

Meningococci are classified into serogroups based on capsular polysaccharides and are further subdivided into serotypes and serosubtypes based on antigenic variants of the outer membrane protein PorB and PorA, respectively [4]. Further molecular typing is based on multi-locus sequence typing (MLST, 7 genes) and sequence analysis of two variable regions (VR1 and VR2) of the class1 *porA* and the *fetA* gene (encoding for an iron-regulated meningococcal OMP) [5;6]. For this molecular typing, a database is hosted at http://neisseria.org/nm/typing containing the sequences of all known MLST profile, *fetA* and VR1/VR2-*porA* sequence types [7].

The most common serogroups causing invasive meningococcal disease are A, B, C, Y and W135. Serogroup B is the most widespread in Europe, followed by serogroup C [2;3]. Untill 2000, serogroup B has always been the main cause of meningococcal disease in Belgium [8;9], but in the late nineties the incidence of serogroup C disease increased considerably and peaked in 2001. In December 2001, because of this increased incidence of serogroup C infections and the higher mortality, the Belgian health authorities decided to finance a mass immunization campaign [10]. Vaccination campaigns were organized at regional level and programmes differed between Flanders (Nord) and Wallonia (South). In Wallonia the campaign targeted only children aged 1–5 years. In Flanders, where a steeper rise of serogroup C disease was reported, the campaign started earlier and was expanded to other age groups, covering the entire cohort 1–18 years by the end of 2004 [11]. Vaccination against serogroup C meningococcal infection was incorporated in the childhood immunization programme from 2002 onwards, with one dose of vaccine at the age of 12–15 months.

We study the evolution of IMD in Belgium over a length of 16 years including an exhaustive vaccination campaign. The results are based on laboratory surveillance of the serogroups, serotypes, serosubtypes and sequence types of meningococcal strains, isolated from patients with IMD, in Belgium during the time period 1997–2012.

## Materials and Methods

In Belgium, cases of IMD are subject to mandatory notification to the regional Health Inspectorate. The Belgian Meningococcal Reference Centre (BMRC) receives isolates from peripheral laboratories on voluntary base to produce national statistics on meningococcal disease. The BMRC exists since the 1960s and has a strong network with the hospitals and health authorities which guarantees a constant surveillance of IMD in Belgium.and covers about 75% of notified cases [9]. There have been no changes in the testing methodology during the study period.

### Bacterial strains and typing

Between January 1997 and December 2012, 2997 strains isolated from patients with invasive meningococcal disease were received at the Belgian Meningococcal Reference Centre (BMRC). Bacteria were grown at 37°C on Columbia Sheep Agar (Oxoid) in a candle air-exhaustion chamber. Serogrouping was performed by slide agglutination with commercial antisera (Becton Dickinson, Sparks, USA). Serotypes and subtypes were determined by whole-cell ELISA with monoclonal antibodies (NIBSC, Potters Bar, UK) [12].

### Molecular analysis

Multi-locus sequence typing (MLST) was done on a subset of 276 isolates from 2000–2002 (representative number of selected strains: serogroup B: one out of three randomly selected, other serogroups: all strains tested) according to the method of Maiden et al. [5] at the European Meningococcal MLST Centre (University of Oxford, UK). All other MLST were carried out using the method of Taha et al. [6] at the BMRC. Between 2008–2010 MLST analysis was performed on one out of three randomly selected serogroup B isolates and all non-serogroup B isolates, between 2011–2012 all strains were analysed. Isolates were assigned to clonal complexes according to the *Neisseria* MLST website (http://pubmlst.org/neisseria/) [7].

Amplification and sequencing of the *porA* and *fetA* genes was carried out as described in Russell et al. [13] and Thompson et al. [14], respectively. The *porA* variable regions, VR1 and VR2 and *fetA* variable regions were assigned using the databases of the *Neisseria*.org website (http://neisseria.org/nm/typing/).

### Data analysis

Incidence rates are calculated based on population data obtained from http://statbel.fgov.be/nl/statistieken/cijfers/bevolking/structuur/leeftijdgeslacht/belgie/ Statistical significance was assessed by calculating incidence rate ratios (IRR) with Poisson distributions. The 95% confidence intervals are indicated between []. Differences in proportions were calculated using the Chi square Mantel-Haenszel test. Calculations were performed with Stata/SE 14.0 software.

## Results

### Epidemiological data and phenotypic characterisation of the strains

**Fig 1:**
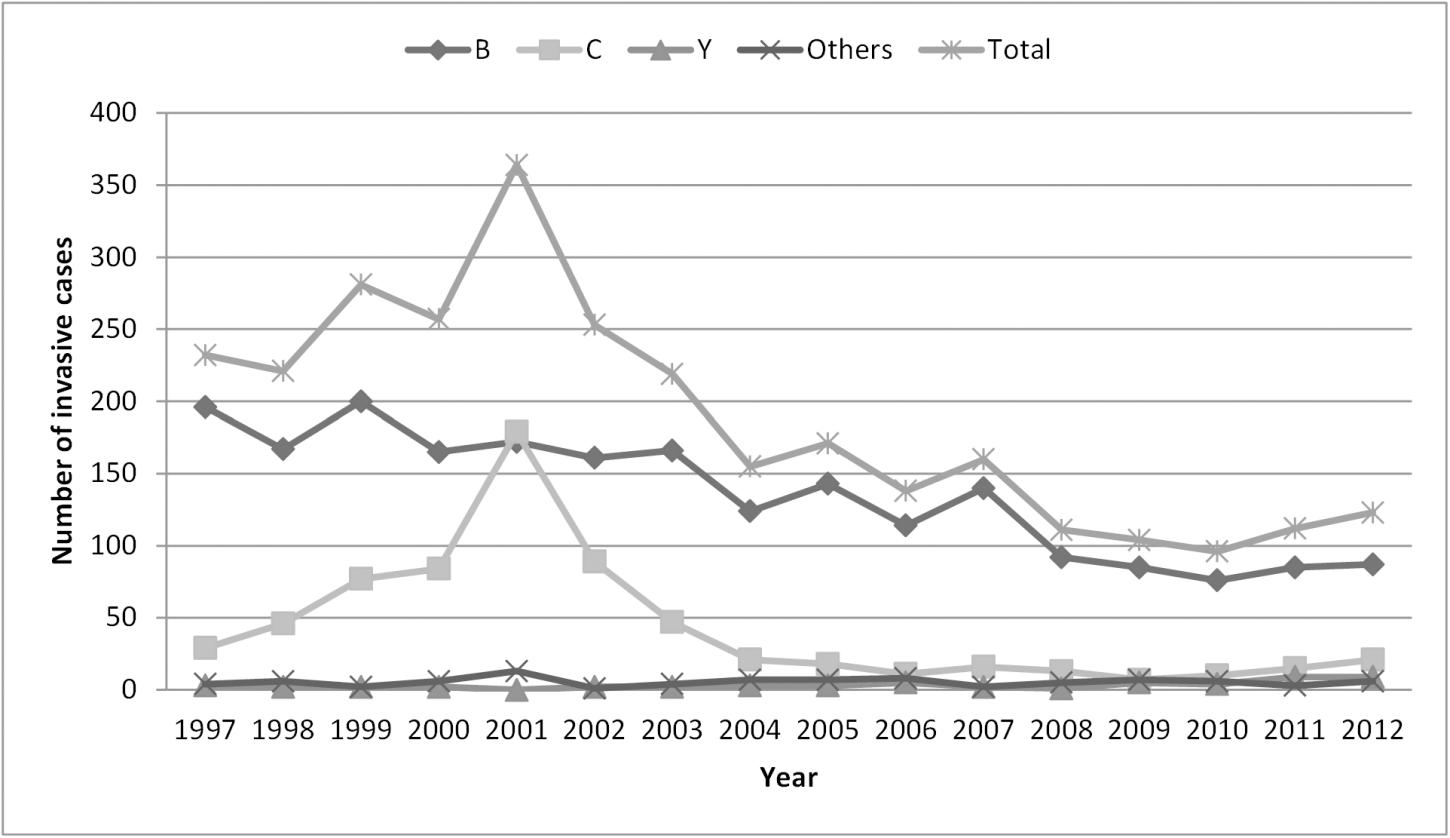
Number of laboratory confirmed invasive meningococcal disease cases in Belgium, 1997–2012. Serogroup C vaccination period 2001–2004.

From 1997 to 2012, isolates from 2997 patients (1556 males, 1429 females, 12 of unspecified gender; sex ratio 1.09) were submitted to the BMRC, the annual number of strains varying between 96 and 364 (Fig 1). Serogroup could be identified for 2983 meningococcal strains (99.5%) and serogroup B was the most common (72.5%), followed by serogroup C (22.8%), serogroup W135 (2.7%). and serogroup Y (1.8%). Other serogroups (A, X and 29E) accounted each for less than 1% of the total typed cases.

From 1997 to 2001, the annual incidence rate of meningococcal disease based on submission of strains to the BMRC increased from 2.28 [2.00–2.59] to 3.55 [3.19–3.93]/100,000 inhabitants (IRR = 1.55 [1.32–1.83]) (Table 1). The annual incidence of serogroup B infections slightly decreased (1.93 [1.68–2.22]/100,000 in 1997, 1.68 [1.43–1.95]/100,000 in 2001, IRR = 0.87 [0.71–1.07]), while that of serogroup C infections increased 6-fold in Belgium, from 0.29 [0.19–0.41] to 1.74 [1.50–2.02]/100,000 inhabitants (IRR = 6.11 [4.13–9.05]). However, marked differences according to regions were observed: serogroup C IMD rose from 0.33 [0.17–0.60] to 1.08 [0.75–1.49]/100,000 in Wallonia (IRR = 3.25 [1.65–6.38]) and from 0.20 [0.11–0.36] to 2.27 [1.90–2.68]/100,000 in Flanders (IRR = 11.15 [6.17–20.12]) with some districts reaching incidence above 5/100,000. Increasing incidence rates were reported in all age groups over the period 1997–2001 especially among infants (from 28.59 [19.68–40.15] to 44.76 [32.71–57.76]/100,000 infants), among children aged 1–4 years (from 14.77 [11.51–18.66] to 22.05 [17.98–26.77]/100,000 children) and among teenagers aged 15–19 years (from 6.74 [4.86–9.11] to 11.23 [8.70–14.20]/100,000 teenagers). An increase in mortality was also observed: the overall case fatality rate (CFR) rose from 4.7% in 1997 to 7.4% in 2001, yet the outcome being only reported in 47% of the cases. The number of fatal infections attributable to serogroup C rose from 2 cases in 1999 to 7 in 2000 and to 22 in 2001 (at this time, CFR was 12.3% (22/179) for serogroup C compared to 3% (5/172) for serogroup B).

**Table 1:** Number of laboratory confirmed invasive Meningococal disease cases in Belgium, 1997–2012: number of cases, number of deaths, case fatality rate (CFR), serogroup distribution, and incidence.

Year	No of	No of	CFR						Overal
	cases	deaths				N (%; No per100,000)			Incidence^a^
				B	C	Y	W135	Other	(No/100,000)
1997	232	11	4.7	196 (84.48%;1.93)	29 (12.5%;0.29)	3 (1.29%;0.03)	4 (1.72%;0.04)	0 (0%;0)	2.28
1998	221	13	5.9	167 (75.57%;1.64)	46 (20.81%;0.45)	2 (0.9%;0.02)	5 (2.26%;0.05)	1 (0.45%;0.01)	2.17
1999	281	16	5.7	200 (71.17%;1.96)	77 (27.4%;0.75)	2 (0.71%;0.02)	1 (0.36%;0.01)	1 (0.36%;0.01)	2.75
2000	257	13	5.1	165 (64.2%;1.61)	84 (32.68%;0.82)	2 (0.78%;0.02)	4 (1.56%;0.04)	2 (0.78%;0.02)	2.51
2001	364	27	7.4	172 (47.25%;1.68)	179 (49.18%;1.74)	0 (0%;0)	10 (2.75%;0.1)	3 (0.82%;0.03)	3.55
2002	253	15	5.9	161 (63.64%;1.56)	89 (35.18%;0.86)	2 (0.79%;0.02)	1 (0.4%;0.01)	0 (0%;0)	2.45
2003	219	9	4.1	166 (75.8%;1.6)	47 (21.46%;0.45)	2 (0.91%;0.02)	3 (1.37%;0.03)	1 (0.46%;0.01)	2.11
2004	155	10	6.5	124 (80%;1.19)	21 (13.55%;0.2)	3 (1.94%;0.03)	5 (3.23%;0.05)	2 (1.29%;0.02)	1.49
2005	171	14	8.2	143 (83.63%;1.37)	18 (10.53%;0.17)	3 (1.75%;0.03)	6 (3.51%;0.06)	1 (0.58%;0.01)	1.64
2006	138	2	1.4	114 (82.61%;1.08)	11 (7.97%;0.1)	5 (3.62%;0.05)	8 (5.8%;0.08)	0 (0%;0)	1.31
2007	160	10	6.3	140 (87.5%;1.32)	16 (10%;0.15)	2 (1.25%;0.02)	1 (0.63%;0.01)	1 (0.63%;0.01)	1.51
2008	111	4	3.6	92 (82.88%;0.86)	13 (11.71%;0.12)	1 (0.9%;0.01)	3 (2.7%%;0.03)	2 (1.8%;0.02)	1.04
2009	104	5	4.8	85 (81.73%;0.79)	7 (6.73%;0.07)	5 (4.81%;0.05)	4 (3.85%;0.04)	3 (2.88%;0.03)	0.97
2010	96	8	8.3	76 (79.17%;0.7)	10 (10.42%;0.09)	4 (4.17%;0.04)	4 (4.17%;0.04)	2 (2.08%;0.02)	0.89
2011	112	8	7.1	85 (75.89%;0.78)	15 (13.39%;0.14)	9 (8.04%;0.08)	1 (0.89%;0.01)	2 (1.79%;0.02)	1.02
2012	123	7	5.7	87 (70.73%;0.79)	21 (17.07%;0.19)	9 (7.32%;0.08)	3 (2.44%;0.03)	3 (2.44%;0.03)	1.11

^a^Population data obtained from: http://statbel.fgov.be/nl/statistieken/cijfers/bevolking/structuur/leeftijdgeslacht/belgie/

Serogroup C vaccination period 2001–2004.

From 2001 to 2004, the vaccination campaign was implemented and the annual incidence rate of meningococcal disease fell from 3.55 [3.19–3.93] to 1.49 [1.27–1.74]/100,000 inhabitants (IRR = 0.42 [0.35–0.51]). Whereas the number of serogroup B infections only slightly decreased, a 88% decrease in serogroup C disease incidence has been observed, from 1.74 [1.50–2.02] to 0.20 [0.13–0.31]/100,000 inhabitants (IRR = 0.12 [0.07–0.18]). The number of reported deaths due to serogroup C also felt from 22 in 2001 to 8 in 2002 and to 4 in 2004. The decrease in serogroup C was markedly in both regions, yet sharper in Flanders (92%) compared to Wallonia (77%). This decrease was observed in all age groups: 0–4 years 91%, 5–19 years 94% and >19 years 78%.

In the late post-vaccination period (2004–2012) a further decline in IMD reported cases was seen and the incidence rate dropped from 1.49 [1.27–1.74] to 1.11 [0.93–1.33] /100,000 inhabitants (IRR = 0.75 [0.59–0.95])., mainly caused by a drop of serogroup B cases from an annual incidence of 1.19 [0.99–1.42]/100,000 in 2004 to 0.79 [0.63–0.97]/100,000 in 2012 (IRR = 0.66 [0.50–0.87]). Furthermore, a slight increase in serogroup Y isolates was observed from 0–3 cases/year between 1997–2004 to 3–9 cases/year between 2005–2012.

Various phenotypes were recovered over the period 1997–2012: the most common were B:4:P1.4 (27%), C:2a:P1.5,2 (6.4%), C:2b:P1.5,2 (3.5%), C:2a:P1.5 (3.2%), B:NT:P1.14 (3.2%), B:15:P1.7,16 (1.8%), B:4:P1.15 (1.8%) and C:2b:P1.1,7 (1.6%).

**Fig 2:**
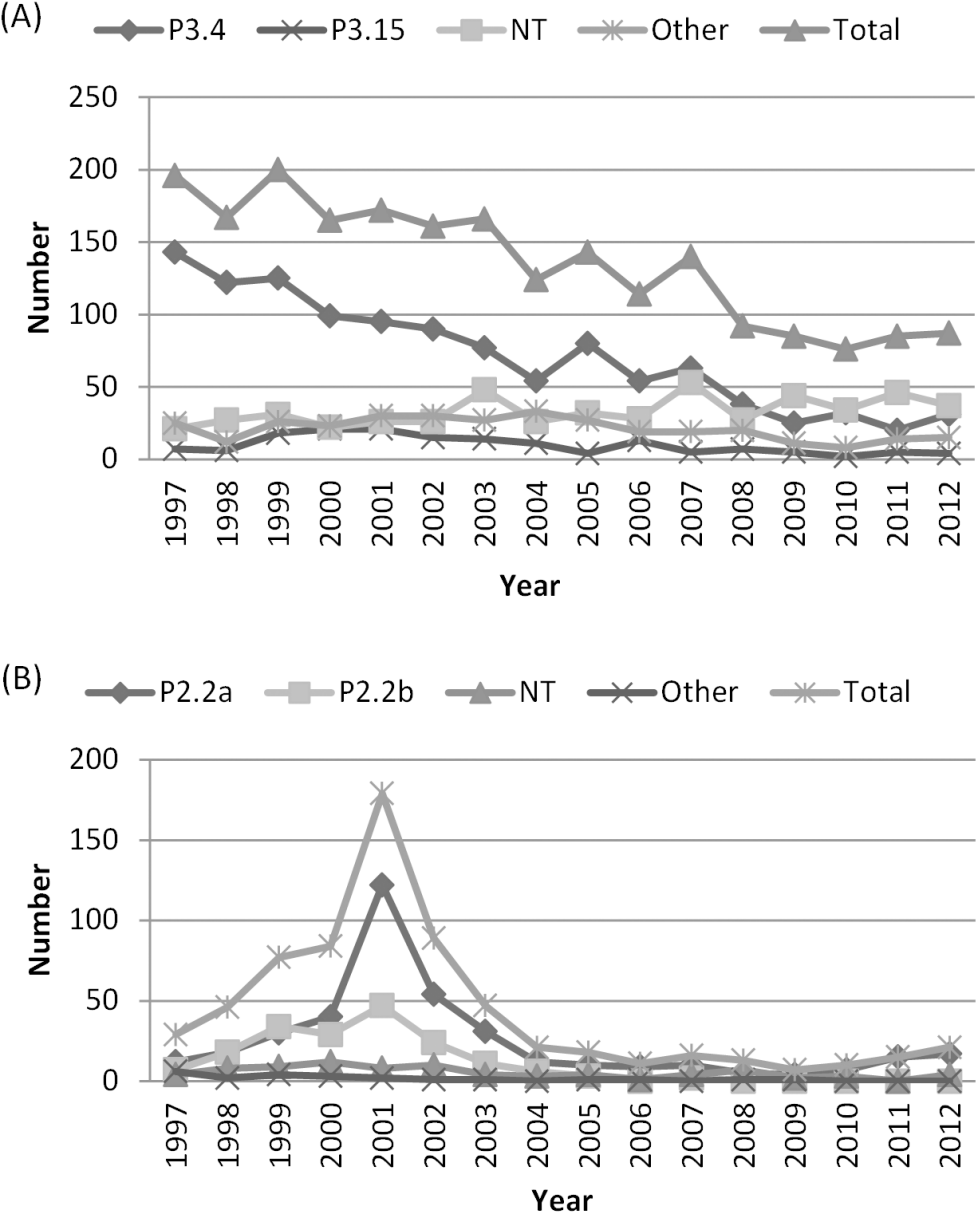
Number of laboratory confirmed invasive meningococcal disease cases per serotype in Belgium, 1997–2012. (**A)**: serogroup B; (**B)**: serogroup C.

The serotype distribution of invasive meningococcal isolates is shown in Table 2. During this period, the serogroup:serotype combinations B:4, C:2a and C:2b together accounted for 57.5% of the serotyped isolates, non-typable (NT) isolates represented 17%. Serotype 2a was the most commonly identified among serogroup C meningococci. However, the increase of serogroup C was first associated with a rise of both serotype 2a and 2b. Serotype 2b was predominant in 1999 and mainly recovered in the province of West-Flanders (especially C:2b:P1.2,5 isolates). But between 2000 and 2001, the number of C:2a isolates tripled (from 40 to 122 strains) and in 2001, serotype 2a was responsible for 68% (122/179) of serogroup C infections. Seventy-three per cent of these infections were detected in Flanders (especially in the province of Antwerp, 30.3%). Serotype 2a remained the major serotype in serogroup C after the vaccination campaign, although at a much lower numbers, while C:2b isolates completely disappeared since 2008 (Fig 2A). Between 2001 and 2004, 14 B:2a:P1.5,2 and 5 B:2a:P1.2 strains were isolated in Flanders (especially in the provinces of East-Flanders and Antwerp), though B:2a meningococci were scarcely detected in the 1990s and hints at a possible capsule switching.

**Table 2:** Number of invasive meningococcal isolates in Belgium per serotype, 1997–2012.

		No of isolates per year															
		1997	1998	1999	2000	2001	2002	2003	2004	2005	2006	2007	2008	2009	2010	2011	2012
B	**P2.2a**	1	0	1	0	7	6	8	1	0	0	0	0	1	0	0	1
	**P2.2b**	2	1	2	4	2	1	4	6	3	4	2	0	0	0	0	1
	**P3.1**	5	4	12	9	6	10	5	9	11	9	3	11	4	5	7	5
	**P3.14**	16	4	9	7	12	7	3	9	8	0	0	0	0	0	0	0
	**P3.15**	7	6	18	21	21	15	14	11	4	13	5	7	5	2	5	4
	**P3.21**	1	3	2	3	3	6	7	8	5	6	14	9	6	3	7	8
	**P3.4**	143	122	125	99	95	90	77	54	80	54	63	38	25	32	20	31
	**NT**	21	27	31	22	26	26	48	26	32	28	53	27	44	34	46	37
	**Total**	196	167	200	165	172	161	166	124	143	114	140	92	85	76	85	87
C	**P2.2a**	12	18	30	40	122	54	31	12	10	9	10	5	4	7	15	17
	**P2.2b**	7	18	34	29	47	24	11	6	3	0	2	0	0	0	0	0
	**P3.1**	0	0	0	0	0	0	0	0	0	0	0	0	0	0	0	0
	**P3.14**	1	0	1	1	0	1	0	0	0	0	0	0	0	0	0	0
	**P3.15**	0	1	0	2	0	0	0	0	0	1	0	0	0	0	0	0
	**P3.21**	2	0	0	0	0	0	1	0	1	0	0	0	1	0	0	0
	**P3.4**	3	1	3	0	2	0	0	0	0	0	0	1	0	0	0	0
	**NT**	4	8	9	12	8	10	4	3	4	1	4	7	2	3	0	4
	**Total**	29	46	77	84	179	89	47	21	18	11	16	13	7	10	15	21
Y	**P2.2a**	0	0	0	0	0	0	0	0	0	0	0	0	1	0	0	0
	**P2.2b**	0	0	0	0	0	0	0	0	0	0	0	0	0	0	0	0
	**P3.1**	0	0	0	0	0	0	0	0	0	0	0	0	1	1	0	0
	**P3.14**	2	0	0	0	0	2	0	0	0	0	0	0	0	0	0	0
	**P3.15**	0	0	0	0	0	0	1	1	1	2	0	0	1	0	0	0
	**P3.21**	0	0	0	0	0	0	0	0	0	0	0	0	0	0	1	0
	**P3.4**	1	1	1	0	0	0	0	1	0	0	0	0	0	1	0	1
	**NT**	0	1	1	2	0	0	1	1	2	3	2	1	2	2	8	8
	**Total**	3	2	2	2	0	2	2	3	3	5	2	1	5	4	9	9
W135	**P2.2a**	0	0	0	1	1	0	0	1	0	1	0	1	1	0	0	2
	**P2.2b**	0	0	0	0	0	0	0	0	0	0	0	0	0	0	0	0
	**P3.1**	0	0	0	0	0	0	0	0	0	0	0	0	0	0	0	0
	**P3.14**	0	0	0	0	0	0	0	0	0	0	0	0	0	0	0	0
	**P3.15**	0	0	0	0	0	0	0	0	0	0	0	0	0	1	0	0
	**P3.21**	0	0	0	0	0	0	0	0	0	0	0	0	1	0	0	0
	**P3.4**	2	0	0	0	0	0	0	0	0	0	0	0	0	0	0	0
	**NT**	2	5	1	3	9	1	3	4	6	7	1	2	2	3	1	1
	**Total**	4	5	1	4	10	1	3	5	6	8	1	3	4	4	1	3

Serogroup C vaccination period 2001–2004.

Serotype 4 was the predominant serotype in serogroup B; however, its frequency steadily decreased from 73% in 1997 to 41% in 2008 (p < 0.01) and continued fluctuating around this level till 2012. On the other hand, within serogroup B the proportion of non-serotypable strains increased from 20% before 2007 to 40% after 2007 (Fig 2B). In serogroup W135 and Y, a majority of isolates were non-typable (mainly W135:NT:P1.3,6, W135:NT:P1.2,5 and Y:NT:P1.5).

### Multi Locus Sequence Typing

**Fig 3:**
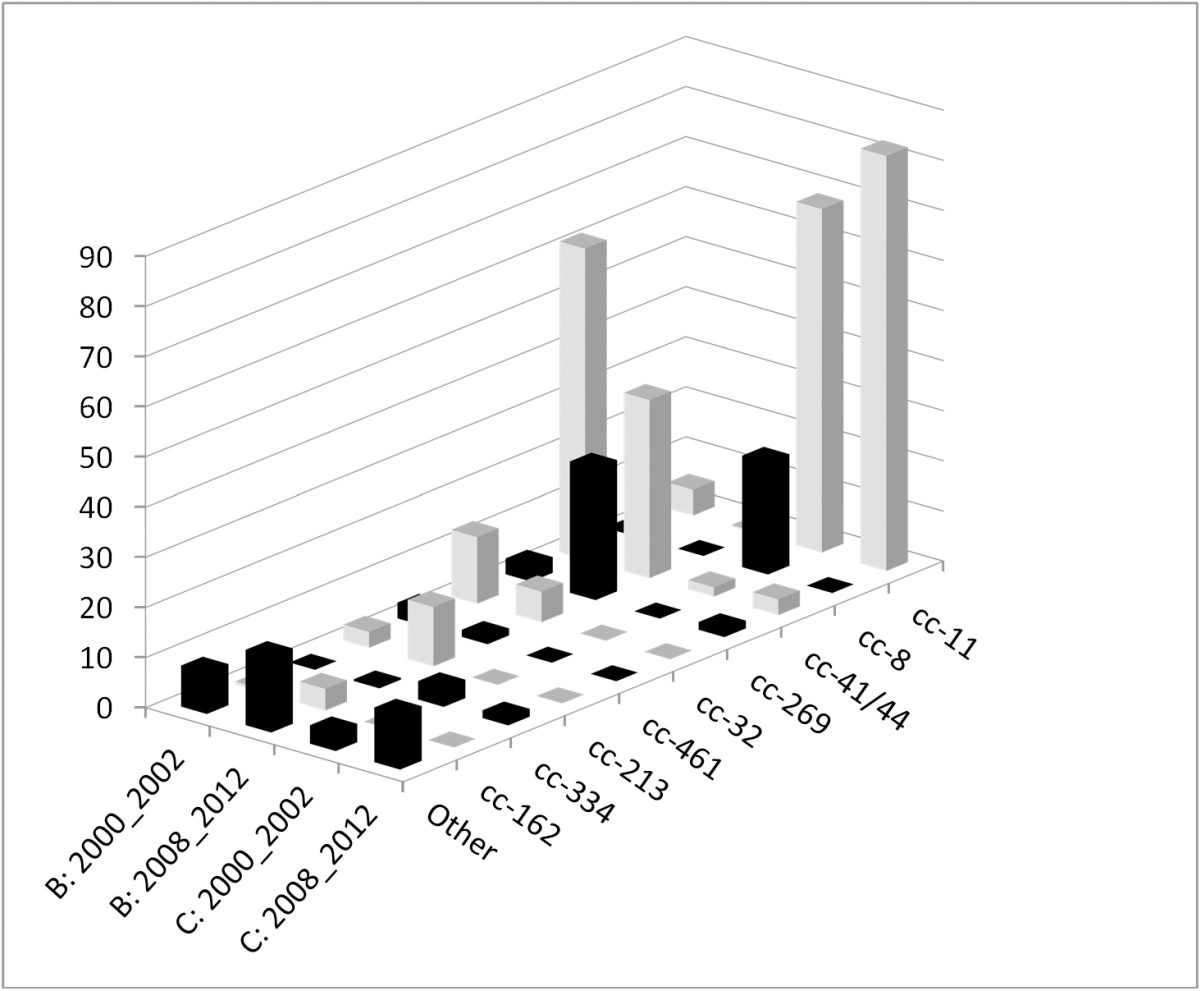
Proportional MLST distribution of serogroup B and C during the epidemic peak (2000–2002) and after the vaccination campaign (2008–2012).

Analysis by multi-locus sequence typing was performed on a representative population of 276 *N*. *meningitidis* strains isolated in 2000–2002, during the peak of serogroup C meningococcal disease, and of 389 *N*. *meningitidis* strains isolated in 2008–2012 (Fig 3).

### Period 2000–2002

One hundred and forty five different sequence types (STs) were identified among 277 tested strains and the most common were ST-11, ST-41, ST-42 and ST-66. Two hundred and twenty nine (83.0% of typed strains) belonged to four hyper invasive complexes: 100 to ST-41/44 complex (lineage III)(36.2%), 82 to ST-11/ET-37 complex (29.7%), 26 to ST-8-complex (cluster A4)(9.4%) and 21 to ST-32/ET-5 complex (7.6%). Seventeen other clonal complexes (ST-269, ST-461, ST-22, ST-213, ST-254, ST-334,…) were identified among 39 isolates. The 8 remaining strains were mostly heterogeneous and not assigned to a clonal complex. Among the 157 serogroup B isolates the distribution of ST complexes showed predominance of ST-41/44complex (62.4%) and ST-32 complex (13.4%). The B:4:P1.4 isolates belonged to the ST-41/44complex which regrouped about 20 phenotypes and exhibited 38 different STs, reflecting its large diversity. The ST-32 complex encompassed mainly the phenotypes B:15:P1.7,16 and B:4:P1.15. Among the 108 serogroup C isolates ST-11 complex (68.5%) and ST-8-complex (22.2%) were predominant. The C:2a isolates responsible for the outbreak belonged to the ST-11 complex and the C:2b strains to the ST-8 complex (mainly to ST-66). The B:2a isolates emerging in 2001 also belonged to the ST-11 complex.

The majority of the serogroup W135 isolates (4/6) belonged to the ST-22 complex, one serogroup W135:2a:P1.5,2 belonged to the ST-11 complex. The serogroup Y isolated belonged to ST23 (1/2) and ST-167 (1/2) complex

### Period 2008–2012

One hundred and three different sequence types (STs) were identified among 390 tested strains and showed a similar distribution across the 2008–2012 period. The most common STs were ST-11, ST-41 and ST-269. Two hundred and sixty-eight (68.9% of typed strains) belonged to four hyper invasive complexes: 102 to ST-41/44complex (lineage III) (26.2%), 75 to ST-269 complex (19.3%), 57 to ST-11/ET-37 complex (14.7%) and 34 to ST-213 complex (8.7%). Twenty-four other clonal complexes were identified among 111 isolates. The 60 remaining strains were mostly heterogeneous and not assigned to a clonal complex. Clonal complex ST-269 consisted mainly of B:NT isolates (89.3%, 67/75), of which 70.1% (47/67) were of serosubtype P1.14. This serosubtype also dominated the ST-213 complex with 29 out of 34 isolates (85.3%). The ST of serogroup Y were heterogeneous but the majority belonged to ST-103 (8/26), ST-167 (4/26) and ST-5436 (6/26) which emerged in 2011.

### FetA, porA VR1/VR2

Since 2009, we analysed the *fetA* allele (n = 401), resulting in 37 profiles, and the *porA* VR1 and VR2 (n = 347), resulting in 67 profiles. The main *fetA* profiles were F1-5 (27.2%), F5-1 (21.2%), F5-5 (10.7%) and F3-3 (9.7%). For *porA*, the most prevalent profiles were (VR1,VR2): 7–2,4 (25.6%), 22,14 (17.6%) and 5,2 (10.7%). The first profile (7–2,4) consisted of serogroup B members, most of them B:4:P1.4 (62.9%) and B:NT:P1.4 (22.5%). Almost all MLST analysed B:7–2,4 belonged to the ST-41/44 complex (93.3%) and showed the *fetA* F1-5 profile (81%). The B:22,14 group was divided in the ST-269 complex (57.4%) and the ST-213 complex (40.7%) with *fetA* allele F5-1 and F5-5, respectively. The C:5,2 and W135:5,2 belonged to the ST-11 complex. Remarkably, the C:5,2 isolates isolated in 2008–2012 almost exclusively showed a F3-3 *fetA* allele, whereas the 2000–2002 epidemic isolates exclusively showed the F3-6 *fetA* allele.

## Discussion

This laboratory-based surveillance during 1997–2012 showed marked changes in the distribution of serogroups.

Since 1991, an increase in incidence of meningococcal disease caused by *N*. *meningitidis* B:4:P1.4 belonging to the lineage III was observed in Belgium [15]. From 1997 onwards, whereas the number of serogroup B disease remained stable, an increasing number of cases due to serogroup C meningococci was reported and became predominant in 2001. This upsurge was chiefly caused by strains C:2a:P1.2,5, C:2a:P1.5 and C:2a:P1.2 belonging to the ST-11/ET-37 complex and was especially marked in Flanders (Antwerp). In the late nineties, increased incidence of serogroup C meningococcal disease caused by this hyper-invasive complex has been observed in several European countries [16]. This situation led the health authorities to introduce routine and catch-up conjugate meningococcal group C vaccination programmes in the UK (1999) [17], in Ireland (2000), in Spain (2000) [18], in Belgium (2001) and in The Netherlands (2002) [19]. In France, local vaccination campaigns were implemented to control outbreaks (e.g. Puy-de-Dome; [20]). Although immunization schedules, targeted population and vaccination coverage differed in all these countries, a marked fall in incidence of meningococcal C disease occurred soon after the vaccination campaign was carried out, suggesting that campaigns were effective. In Belgium, where the campaign ended in December 2004, vaccination coverage was high for the 0–4 years age group targeted by the campaign in both Wallonia and Flanders (93% and 94%, respectively)[21]. In Flanders, where the rise in serogroup C incidence was more substantial, the extended vaccination campaign (target group 0–18 years) resulted in a higher vaccination coverage in the 5–19 years age group (63%) compared to Wallonia (30%)[21]. Already one year after the beginning of the Belgian vaccination campaign, a decrease by 50% in incidence of meningococcal C disease was noticed and from 2001 to 2004, a 88% decrease in serogroup C disease was observed. In Flanders the decline of serogroup C was sharper (92%) compared to Wallonia (77%). This decline was observed in all age groups, with the same magnitude in the population targeted by the vaccination than in the unvaccinated population:the age cohort 0–4 years-that has been nationally targeted for immunization (94% vaccine coverage)- showed a 91% decrease of serogroup C between 2001 and 2004 and the adults (above 19 years)-that have not been immunized in any region of the country- displayed a 78% decline of serogroup C in the same period. Although the vaccination coverage differed significantly for the 5–19 year age group between Flanders and Wallonia, serogroup C IMD incidences were significantly reduced in both regions by 2004 (0.20 [0.02–0.9] and 0.30 [0.04–1.1]/100,000 5–19 year olds, respectively). However, the difference in targeted population resulted since 2008 in a remarkable disparity in incidence of serogroup C in Flanders (0–0.1/100,000) versus Wallonia (0.1–0.3/100,000) (OR_Mantel-Haenszel_ = 2.78, p < 0.001). These observations may be explained by a natural decline in the incidence of the serogroup C, but also by herd immunity and population dynamics. The UK Meningococcal carriage study showed that the success of the vaccination campaign could mainly be attributed to herd protection due to the large effect of the vaccination on serogroup C carriage [22]. The carriage study showed that the epidemic clone C:2a: ST-11/ET-37 was especially affected by the vaccination. During carriage meningococci normally down regulate the expression of their capsule. The epidemic clone C:2a: ST-11/ET-37 (and to a lesser extend C:2b: ST-8) however showed high rates of capsule expression (81% and 50%, respectively) [22]. A Dutch study demonstrated similar effects, with a reduction of the proportion of disease caused by ST-11/ST-8 from 94% to 81% among serogroup C IMD [23]. The higher rate of capsule expression observed by ST-11/ET-37 compared to ST-8 led to the assumption that the herd effect might be greater on ST-11/ET-37 [24]. Our study showed a complete disappearance of ST-8 complex, yet a persistence of ST-11/ET-37 of 90–100% of serogroup C IMD since 2008. For the period 2007 till 2012, the occurrence of the different clonal complex can be compared between Belgium and neighbouring countries, based on the data from EMERT. The ST-11/ET-37 complex appearance was higher in France (24.3%) and Germany (20.7%), yet much lower in the Netherlands (4.7%) and England/Wales (2.7%) than in Belgium (14.7%). These higher rates can be explained by the different vaccination strategies (i.e. only local vaccination in case of outbreak in France [20] and late routine infant vaccination since 2006 without catch up in Germany [25], compared to the extensive campaigns in England/Wales [17] and the Netherlands [19]). It has become clear that protective antibody titers wane already one year after infant vaccination. How long the herd protection will last is unknown. Simulation models in the UK predict a carriage protection of 3 to 10 years after the massive vaccination campaign [24].Our data shows that targeting a smaller number of cohorts, based on differing epidemiology, can have similar short term effects on disease incidence, yet may result in long term persistence. These factors are important on the future evaluations of vaccine cost-effectiveness and vaccination strategies.

Although data on vaccination status were incomplete, no vaccine failure was explicitly reported. On the other hand it is worthy to note that from 2001 till 2004 an emergence of B:2a:P1.5,2 and B:2a:P1.2 strains was detected in Flanders, though B:2a meningococci were scarcely detected in the 1990s. All these strains belonged to the ST-11 complex with PorA (VR1, VR2): 5,2 and FetA: F3-6, as seen for the serogroup C counterpart. Therefore these strains could be the result of a capsule-switching genetic mechanism and could have their origin in C:2a:P1.2,5 and C:2a:P1.2 strains. During the period 2008–2012 this phenotype/genotype was detected only once among serogroup B. Similar observations have been reported in other European countries [26–28]. As reported in other European countries, there was a slight increase in serogroup Y since 2009, yet of clonal complexes not linked with serogroup C.

Various phenotypes were identified during 1997–2012 showing that the population of meningococci circulating in Belgium was heterogeneous. However, the analysis of isolates by MLST showed that meningococcal disease in Belgium before 2003 could mainly be attributed to strains belonging to four major hypervirulent clonal complexes (ST-41/44 complex/Lineage III, ST-11/ET-37 complex, ST-8 complex/Cluster A4 and ST-32/ET-5 complex), all of which account for elevated levels of disease worldwide. The period from 2008 till 2012 showed a different clonal complex pattern. The change can be partly attributed to the serogroup C vaccination (48.3% and 100% decrease for ST-11/ET-37 complex and ST-8 complex/Cluster A4, respectively). The rise of ST-269 complex (from 2.2% to 19.3%) is mainly caused by an endemic serogroup B cluster in Flanders [29].

In conclusion, this study highlights the influence of different regional IMD epidemiology on the regional vaccination strategies and the effect of such campaigns on the circulating strains.

## Supporting Information

S1 Dataset Raw data file. (XLSX)
